# Microfocused Ultrasound With Visualization for Body Indications: A Global Expert Consensus on Best Practices for Treatment of the Abdomen and Arms

**DOI:** 10.1111/jocd.70449

**Published:** 2025-09-25

**Authors:** Frank Lin, Vasanop Vachiramon, Gabriela Casabona, Jessie Cheung, Ana Lúcia Gonzaga da Cunha, Tatjana Pavicic, Julieta Spada, Sabrina Fabi

**Affiliations:** ^1^ Monash University Clayton Victoria Australia; ^2^ Division of Dermatology Faculty of Medicine Ramathibodi Hospital, Mahidol University Bangkok Thailand; ^3^ Ocean Clinic Marbella Málaga Spain; ^4^ Cheung Aesthetics & Wellness Willowbrook Illinois USA; ^5^ Albert Einstein Teaching and Research Center São Paulo Brazil; ^6^ Private Practice for Dermatology & Aesthetics Dr. Tatjana Pavicic Munich Germany; ^7^ Spada Dermatology & Aesthetics Buenos Aires Argentina; ^8^ University of California San Diego California USA

**Keywords:** body contouring, consensus, energy‐based device, MFU‐V, real‐time visualization, real‐world evidence, skin laxity

## Abstract

**Background:**

Microfocused ultrasound with visualization (MFU‐V) is well established for noninvasive lifting of the face, neck, and décolleté. U.S. FDA clearance was recently granted for treatment of the abdomen and upper arms, supported by favorable safety data and emerging evidence of efficacy of its use to address skin and soft tissue laxity in body regions. Guidance on best practices for these additional indications is desirable.

**Aims:**

This consensus aims to provide evidence‐based and practice‐informed recommendations supporting safe, effective, and reproducible MFU‐V treatments for the additional body indications.

**Methods:**

An international, multidisciplinary panel of aesthetic experts developed consensus recommendations for MFU‐V treatment of additional body areas based on clinical evidence, expert experience, and structured virtual discussions.

**Results:**

This global expert consensus affirms MFU‐V as a safe and effective noninvasive modality for treating mild to moderate skin and soft tissue laxity in the abdomen and upper arms. Three key domains were identified as critical to optimizing outcomes: patient selection, treatment planning, and expectation management. Ideal candidates are those with mild to moderate laxity and target tissues located at treatable depths. Real‐time ultrasound imaging was emphasized for accurate targeting, anatomical safety, and treatment precision. The panel developed practical treatment protocols for the abdomen and upper arms, including recommended treatment zones, line densities, and transducer depths tailored to tissue characteristics.

**Conclusion:**

Safe and effective MFU‐V outcomes rely on real‐time ultrasound visualization, thoughtful patient selection, expectation management, and anatomically guided treatment. Continued research is needed to refine protocols and guide emerging body applications.

## 1 Introduction

Microfocused ultrasound with visualization (MFU‐V; Ultherapy, Ulthera System; Ulthera Inc., Raleigh, NC, USA, a company of the Merz Aesthetics group) is widely regarded as the gold standard among energy‐based devices (EBDs) for noninvasive skin and soft tissue lifting [1, 2]. It remains the only U.S. Food and Drug Administration (FDA)‐cleared noninvasive EBD that integrates real‐time ultrasound imaging (DeepSEE technology), allowing visualization of tissue structures up to 8 mm in depth [3]. Ultherapy PRIME (MFU‐V 2.0), the next‐generation system, builds on the established efficacy and safety of earlier technology and incorporates enhancements in real‐time imaging, ergonomics, and treatment delivery to support more precise and comfortable treatments. The device delivers focused ultrasound energy to specific tissue depths (i.e., 1.5, 3.0, and 4.5 mm), creating discrete thermal coagulation points at optimal temperatures (60°C–70°C) that stimulate neocollagenesis and elastogenesis while preserving the epidermis, ultimately leading to progressive tissue lifting [4].

MFU‐V is currently FDA‐cleared for lifting of the brow, submental area, and neck, and for improving fine lines and wrinkles on the décolleté [3]. Its safety and efficacy in these anatomical regions have been demonstrated in more than 100 peer‐reviewed publications [2, 5, 6, 7, 8]. The capacity for precise energy delivery at multiple depths, guided by real‐time visualization, forms the basis of its efficacy and safety profile [2, 6]. Given individual variability in skin and soft tissue profile due to factors such as age, sex, and body composition, the ability to visualize tissue layers is critical for individualized treatment planning [9]. By identifying target tissue layers (superficial and mid dermis, deep dermis, and fibromuscular planes, including components of the superficial musculoaponeurotic system), operators can precisely deliver focused treatment without expending energy on nontarget tissues, while avoiding critical structures such as blood vessels and bones [1]. Although a detailed comparison of MFU‐V with non‐visualization EBDs is beyond the scope of this paper, readers are referred to the reviews by Pavicic et al. [6] and Park et al. [2] for comprehensive discussions of these technologies.

Clinical evidence on safety and efficacy from studies on MFU‐V treatment in other body areas provided support for the recent FDA clearance (2025) of MFU‐V for treating the abdomen, as well as the anterior and posterior arm. Table 1 summarizes these supporting clinical studies, which formed the basis of the recommended treatment protocols presented in this paper.

**TABLE 1:** Summary of clinical studies to support the use of MFU‐V for the expanded indications of abdomen and arms.

Publication	Study design	Sample size (evaluable patients)	Age	Area(s) of body treated	Transducer(s) (MHz‐mm focal depth); total treatment lines	Results summary
Lin (2020) [10]	Prospective clinical study	20 females between 6 and 24 months postpartum	Mean age: 32.7	Abdomen	DS 4–4.5; 380 DS 7–3.0; 380 DS 10–1.5; 380	*Clinician‐reported ASLS* 6 months post treatment, mean ASLS score improved from 2.05 to 1.1 for evaluator 1 and 1.9 to 0.85 for evaluator 2 *IGAIS* At 6 months, 40% of patients were assessed as being “very improved,” 50% “improved,” and 10% “unchanged” *SGAIS* At 6 months, 10% of patients regarded the result as “exceptional” improvement, 60% of patients were “very improved,” 25% of patients were “improved,” and 5% were “unchanged” *Patient‐reported ASLS* At 6 months, mean ASLS score improvement of 1.15 grades with 25% of patients reporting a 2‐grade improvement, 65% of patients reporting a 1 grade improvement, and 10% of patients reporting no improvement *SSRS* At 6 months, 35% of patients reported a 3 grade improvement, 30% reported a 2 grade improvement, 25% reported a 1 grade improvement, and 10% reported no change Mean SSRS improvement for the cohort was 1.9 grade overall at 6 months *Patient satisfaction survey* Patients indicated that, on average, they felt 26% less overweight, 23% less embarrassed, 16.5% less self‐conscious, and 23% less bothered by the appearance of their abdomen. They also indicated that they were on average 30.5% happier with their appearance 6 months after their treatment *Histology* Histological examination of pretreated tissue showed increased total collagen, increased number and thickness of fibrous septae, and no change in fat cells within pretreated tissue compared with the control *Safety* No significant AEs were recorded. At 2 weeks after treatment, 17 of the 20 patients reported mild postprocedural pain, which resolved after a mean duration of 4 days. One patient reported skin welting, which fully resolved after 2 weeks requiring no further intervention
Vachiramon et al. (2020) [11]	Prospective, single‐blinded, randomized controlled study	28 females with abdominal skin laxity	Mean age: 43.3	Abdomen	Single plane: DS 4–4.5; 200 Dual plane: ○ DS 4–4.5; 100 ○ DS 7–3.0; 100	Physician‐rated improvement score Single‐plane treatment: The mean improvement scores in single‐plane treatment were 3.03 (±1.26), 3.43 (±1.35), 2.18 (±0.86) at 1‐, 3‐, and 6‐month follow‐ups, respectively. Dual‐plane treatment: The mean improvement scores were 3.11 (±1.23), 3.39 (±1.34), and 2.02 (±0.79) at 1‐, 3‐, and 6‐month follow‐ups, respectively. Patients who had undergone childbirth: Mean improvement scores at 1‐month follow‐up in subjects were 3.89 (±1.45) and 4.11 (±1.27) for single‐plane treatment and dual‐plane treatment, respectively. Improvement scores were increased at 3‐month follow‐ups but trended downwards at 6‐month follow‐ups in single‐plane and dual‐plane treatments. Patients who never gave birth: Mean improvement scores were 2.63 (±0.96) for single‐plane treatment and 2.63 (±0.90) for dual‐plane treatment. Improvement scores were increased at 3‐month follow‐ups but trended downwards at 6‐month follow‐ups in single‐plane and dual‐plane treatments. *Patient‐rated improvement score* Single‐plane treatment: The mean improvement scores in single‐plane treatment were 4.69 (±2.30), 5.26 (±2.32), and 3.46 (±3.18) at 1‐, 3‐ and 6‐month follow‐ups, respectively. Dual‐plane treatment: The mean improvement scores at 1‐, 3‐, and 6‐month follow‐ups were 4.61 (±2.29), 5.14 (±2.40), and 3.44 (±3.24). Patients who had undergone childbirth: At 1‐month follow‐up, improvement scores in subjects who had undergone childbirth were 5.71 (±2.42) and 5.8 (±2.10) for single‐plane treatment and dual‐plane treatment, respectively. Improvement scores were increased at 3‐month follow‐ups but trended downwards at 6‐month follow‐ups in single‐plane and dual‐plane treatments. Patients who never gave birth: At 1‐month follow‐up, the improvement scores were 4.20 (±2.13) for single‐plane treatment and 4.05 (±2.20) for dual‐plane treatment. Improvement scores were increased at 3‐month follow‐ups but trended downwards at 6‐month follow‐ups in single‐plane and dual‐plane treatments. *Safety* No unanticipated AEs. Erythema and edema were noted in all treated sites, which resolved spontaneously within 1–2 weeks. Bruising was observed along local anesthesia injection sites, which also resolved spontaneously within 10 days. Skin tenderness was observed by all patients on both treatment sides, which spontaneously subsided in 2 weeks without any treatment
Sasaki and Tevez (2012) [12]	Prospective, open‐label, nonrandomized trial	6 subjects	30–72 years	Abdomen	DS 4–4.5; 720 DS 7–3.0; 720 DS 10–1.5; 720	*IGAIS* 5 patients (83%) showed improved skin appearance, with 4 patients (66%) having a moderate improvement, 1 patient (17%) having a mild improvement, and 1 patient (17%) having no improvement *SGAIS* 5 patients (83%) showed improved skin appearance, with 3 patients (50%) having a moderate improvement, 2 patients (33%) having a mild improvement, and 1 patient (17%) having no improvement *Safety* No permanent or unexpected AEs were observed. Erythema was observed immediately after treatment and dissipated within a few hours
Rokhsar and Schnebelen (2015) [13]	Prospective open‐label clinical trial	16 female subjects	35–65 years Mean age: 54	Outer arms	DS 4–4.5 or DS 7–4.5; 125 and DS 7–3.0; 120	*Masked photo assessment* 9 subjects (56%) showed aesthetic improvements in the Ulthera‐treated areas at 90 days *PGAIS* 94% of subjects achieved aesthetic improvement at both 90 and 180 days *SGAIS* 83% and 81% of subjects noted improvement in elbow appearance at 90 days and 180 days, respectively *Patient satisfaction survey* 90‐day follow‐up: 72% indicated that they were very satisfied or satisfied with the aesthetic results they achieved 180‐day follow‐up: 50% of subjects indicated they were satisfied with the results *Safety/risks* No treatment related AEs. 1 AE of hypertension (nontreatment related)
Vachiramon et al. (2021) [14]	Randomized, single‐blinded, controlled study	27 patients with upper arm laxity	Mean age: 43.6	Outer arms	Single plane: DS 4–4.5; 200 Dual plane: ○ DS 4–4.5; 100 ○ DS 7–3.0; 480	*Arm circumference* A modest reduction of arm circumferences was observed on both treatment sides when compared with baseline Single plane: Mean arm circumferences were 28.37 (±2.76) cm, 27.83 (±2.63) cm, 27.77 (±2.49) cm, and 27.79 (± 2.48) cm for baseline, 1, 3, and 6 months, respectively Dual plane: Mean arm circumferences were 28.26 (±2.47) cm, 27.72 (±2.55) cm, 27.71 (±2.48) cm, and 27.90 (±2.40) cm at 6 months *Physician‐rated mean improvement scores* Single plane: Mean improvement scores were 2.22 (±1.05) at 1 month, 2.70 (±1.32) at 3 months, and 1.61 (±0.79) at 6 months Dual plane: Mean improvement scores were 2.00 (±1.00) at 1 month, 2.56 (±1.05) at 3 months, and 1.39 (±0.54) at 6 months Single‐plane treatment was significantly superior in comparison with dual‐plane treatment from the physician's perspective (*p* < 0.05) *IASLSS* Baseline IASLSS of both single‐plane and dual‐plane treatment was similar with 24 (88.9%) from each group having had IASLSS of two and three [3] patients from each group having had IASLSS of 1 (11.1%) Throughout the study, IASLSS followed the same trend as the improvement scores, where most patients showed a reduction in severity score at 1‐ and 3‐months follow‐ups, while at 6 months there was a reverse trend (increasing severity) toward baseline. However, the overall severity at 6 months was still lower than that at baseline *Patient‐rated mean improvement scores* Single‐plane treatment: Mean improvement scores were 3.63 (±2.63), 3.69 (±2.83), and 1.90 (±2.51) for 1, 3, and 6 months, respectively Dual‐plane treatment: Mean improvement scores were 3.5 (±2.57), 3.22 (±2.59), and 1.78 (±2.48) for 1‐, 3‐, and 6‐months follow‐up, respectively Although single‐plane treatment received slightly higher scores in patient‐rated improvement scores, the difference was not statistically significant (*p* = 0.462) *Safety* No unexpected AEs
Sasaki and Tevez (2012) [12]	Prospective, open‐label, nonrandomized trial	44 adults	30–72 years	Inner arms	DS 4–4.5; 480 DS 7–3.0; 480	*IGAIS* 40 patients (91%) showed improved skin appearance, with 31 patients (70%) having a moderate improvement, and 9 patients (20%) having a mild improvement *SGAIS* 37 patients (84%) showed improved skin appearance, with 30 patients (68%) having a moderate improvement, and 7 patients (15%) having a mild improvement *Safety* No permanent or unexpected AEs were observed. Erythema was observed immediately after treatment and dissipated within a few hours
Alster and Tanzi (2012) [15]	Prospective, open‐label trial	12 adult women	44–66 years Mean age: 54.8	Inner arms	Single plane: DS 4–4.5; 60–230 (mean 160) Dual plane: ○ DS 4–4.5; 60–230 (mean 160) ○ DS 7–3.0; 90–235 (mean 153)	*Global clinical improvement scores via physician photo assessment* Single plane: At 3 months, treated patients showed a mean improvement score of 1.83 out of 4, and at 6 months patients showed a mean improvement score of 2.05 out of 4 Dual plane: At 3 months, patients showed a mean improvement score of 1.92 out of 4, and at 6 months patients showed a mean improvement score of 2.25 out of 4 *Safety* No unexpected adverse events. AEs were limited to transient erythema and tenderness in all patients and occasional localized bruising in the arm region
Darji et al. (2024) [16]	Prospective, single‐center, blinded, split body, randomized clinical study	14 adult women	Mean age: 59.5	Inner arms	DS 4–4.5; 180–242 (mean 213) DS 7–3.0; 190–240 (mean 207)	*Arm diameter and circumference* No significant change in arm diameter or circumference. *Skin crepiness/laxity score* Subjects treated with the MFU‐V showed crepiness/laxity scores of ~2.6, ~1.9, and ~1.75 at baseline, 30 days, and 90 days, respectively (lower scores are better). *SGAIS* At day 90, 8 patients (57%) saw improvement. *Safety* No unexpected AEs.

Abbreviations: AE, adverse events; ASLS, Abdominal Skin Laxity Scale; IASLSS, Investigator Assessment Skin Laxity Scoring System; IGAIS, Investigator Global Aesthetic Improvement Scale; MFU‐V, microfocused ultrasound with visualization; SGAIS, Subject Global Aesthetic Improvement Scale; SSRS, Subject Self‐Rating Scale.

This global expert consensus integrates available clinical evidence with perspectives from real‐world practice to provide evidence‐based guidance for the use of MFU‐V in these additional body areas. It outlines optimal treatment zones and protocols, discusses patient selection criteria, and highlights practical considerations for safe and effective MFU‐V treatment for body indications.

## 2 Methods

An international group of eight clinical experts convened in April 2025 in a series of virtual meetings to establish a consensus on the safe and effective use of MFU‐V for additional body indications (abdomen and arms). All panelists had extensive relevant clinical expertise, including a minimum of 10 years of experience with MFU‐V, at least 5 years of experience treating body areas, and authorship/coauthorship of at least three peer‐reviewed publications supporting its use, best practices, and/or innovations. The panel comprised dermatologists and plastic surgeons from Argentina, Australia, Brazil, Germany, Spain, Thailand, and the United States.

The panel's consensus, informed by both the currently available published evidence and the panel's collective clinical experience, addresses optimal treatment principles and protocols, appropriate patient selection, and key practical considerations for safe and effective application of MFU‐V to the abdomen and arms. Consensus on recommendations was determined based on levels of agreement among panelists. Agreement thresholds were categorized as follows: strong consensus (> 95% agreement); consensus (> 75%–95% agreement); majority consent (> 50%–75% agreement); and no majority consent (≤ 50% agreement).

## 3 Results

The consensus guidelines proposed by the expert panel are summarized in Table 2.

**TABLE 2:** Consensus statements.

**General statements**
1. MFU‐V (Ultherapy PRIME, Ultherapy) is currently the only FDA‐cleared noninvasive energy‐based device that incorporates real‐time visualization to accurately target collagen‐rich and elastin‐rich tissue layers at multiple depths to treat skin and soft tissue laxity
2. The real‐time visualization capability of MFU‐V enables personalized treatment planning and enhances procedural safety across diverse patient profiles through delivery of consistent and precise energy based on anatomical visualization
3. A reliable body of scientific and clinical evidence demonstrates that MFU‐V is effective and safe in rendering reproducible outcomes in the treatment of skin and soft tissue laxity in the face/neck/décolletage, as well as other body regions, including the abdomen, arms, buttocks, and knees
**Treatment of the abdominal region**
4. The literature shows that the use of MFU‐V for treatment of the lower abdomen and peri‐umbilical area is associated with improved skin and soft tissue laxity and positive patient satisfaction. Effects can last for 6 months or longer after maximal treatment effects are observed
5. Real‐time visualization should be used to guide transducer selection to match the depth of the target tissue layers, particularly the fascia and dermis. Treatment at three focal depths (4.5, 3.0, and 1.5 mm) is optimal for improving abdominal skin and soft tissue laxity, though patient selection should account for tissue layer distribution and tolerability
6. Recommended treatment regimen for lower abdomen (area bounded by the umbilicus, the hip bones, and the pubic area), assuming implementation of a dual‐depth strategy: Grid of 12 (twelve) 2 × 4 cm rectangular columns A median of 720 treatment lines Recommended treatment regimen for peri‐umbilical area (area immediately surrounding, but excluding, the umbilicus), assuming implementation of a dual‐depth strategy: Grid of 12 (twelve) 2 × 4 cm rectangular columns (excludes the umbilicus) A median of 720 treatment lines The number of lines may be increased or decreased depending on the patient profile (e.g., BMI or body size) and treatment needs. A cautious approach should be taken when treating individuals with a history of hernia in the abdominal region, prior surgical interventions (e.g., liposuction, abdominoplasty, or C‐section), body piercings in the abdominal area, or those with low BMI
**Treatment of the upper arm**
7. The literature shows that the use of MFU‐V for treatment of the anterior and posterior upper arm region is associated with noticeable skin and soft tissue laxity improvement. Effects can last for 6 months or longer after maximal treatment effects are observed
8. For both the anterior and posterior arms, treatment should be confined to the middle third, between the axilla and elbow, avoiding the deeper tissues of the arm, which contain major neurovascular structures
9. Real‐time visualization should be used to guide transducer selection to match the depth of the target tissue layers, particularly the fascia and dermis. Depending on the patient's arm profile (i.e., tissue layer distribution/composition), a dual or triple depth treatment approach (1.5, 3.0, and/or 4.5 mm) may be used to optimize outcomes. Although the 4.5 mm transducer depth is rarely used for the anterior arm, where the fascia are typically more superficial, it may be beneficial for some patients in whom it can be confirmed by visualization that the depth of the fascia are aligned with the 4.5 mm transducer depth
10. Recommended treatment regimen per arm for anterior region, assuming implementation of a dual‐depth strategy: Grid of 6 (six) 2 × 4 cm rectangular columns A median of 240 treatment lines Recommended treatment regimen per arm for posterior region, assuming implementation of a dual‐depth strategy: Grid of 6 (six) 2 × 4 cm rectangular columns A median of 240 treatment lines The number of lines may be increased or decreased depending on the patient profile (e.g., BMI or body size) and treatment needs. Care should be taken when treating the anterior arm to avoid the neurovascular bundle structures on the medial aspect of the arm
**Treatment of other body regions**
11. The safety and effectiveness of MFU‐V for the treatment of skin and soft tissue laxity in other body regions, such as the knees and buttocks, is supported by multiple studies that utilized a range of protocols (varying treatment lines and transducer depths). Further clinical experience and evidence are desired to define suitable treatment parameters for these and other emerging body regions
**Safety statements**
12. The practitioner's knowledge of anatomy and use of real‐time visualization should be used to guide energy delivery and avoid nontarget tissues, especially neurovascular structures or joint capsules, such as those near the axilla and olecranon in upper arm treatments
13. Caution is advised when treating patients with low BMI or those with a history of substantial weight loss. The presence of lower levels of adipose tissue or stretched anatomical structures may increase the risk of injury to neurovascular structures, particularly in areas such as the upper arms. In these cases, real‐time visualization is particularly important for assessing tissue depth and ensuring precise, safe energy delivery
**Conclusion statements**
14. Clinical experience and emerging evidence support the effectiveness of MFU‐V in improving skin and soft tissue laxity across multiple body areas, including the abdomen, arms, buttocks, and knees, when treatment is appropriately tailored to the patient's anatomical characteristics and aesthetic needs
15. MFU‐V demonstrates a favorable safety profile for body treatments when applied using recommended protocols, with good tolerability consistently reported across body regions and patient types. Effective use of visualization is imperative for the treatment of specific anatomical areas, such as the upper arms, particularly in individuals with low BMI or a history of significant weight loss

Abbreviations: BMI, body mass index; FDA, Food and Drug Administration; MFU‐V, microfocused ultrasound with visualization.

### 3.1 General Considerations for MFU‐V in Body Indications

The panel unanimously agreed that MFU‐V is uniquely positioned among noninvasive treatment modalities for skin and soft tissue laxity due to its real‐time visualization capability and ability to deliver precisely targeted energy at multiple tissue depths (Table 2). This consensus is supported by robust scientific and clinical evidence demonstrating the efficacy and safety of MFU‐V, with reproducible outcomes across both facial and body regions [2, 5, 10, 11, 12, 13, 14, 15, 16]. The panel highlighted three essential considerations for optimizing outcomes of MFU‐V treatment in body regions: careful patient selection, comprehensive assessment, and effective management of patient expectations.

#### 3.1.1 Ideal Patient Selection

Panelists agreed that ideal candidates for MFU‐V body treatment are individuals with mild to moderate skin or superficial soft tissue laxity, with sufficient target tissue at treatable depths. Particular benefits were highlighted for addressing postpartum abdominal laxity, and for individuals who decline or are not candidates for surgical interventions such as abdominoplasty. Key clinical challenges identified include variability in tissue characteristics across individuals and difficulty in distinguishing between tissue laxity and adiposity, especially in older individuals and those with higher body mass index (BMI). Additional factors such as age‐related decline in collagen quality and loss of muscle mass (e.g., sarcopenia) were noted to diminish treatment response, especially in postmenopausal women.

#### 3.1.2 Patient Assessment

As with MFU‐V treatments for the face/neck/décolleté, a comprehensive evaluation to confirm patients' suitability for MFU‐V body treatment was considered essential. This evaluation should incorporate a detailed review of the patient's medical, surgical, and aesthetic history, thorough clinical examination, and real‐time ultrasound imaging. Initial visual inspection and manual techniques (e.g., skin pinch tests) may be used for preliminary assessment of laxity. Additionally, some panel members proposed establishing a standardized multi‐grade clinical laxity scale to enhance consistency. With MFU‐V, real‐time visualization facilitates precise evaluation by allowing operators to clearly define tissue composition and discern patient‐specific differences in tissue layer profile and thickness. Treatable structures (e.g., dermis, superficial fascia) are readily distinguished from non‐treatable tissues (e.g., muscle, deep adipose tissue, deep fascia) that either respond poorly to energy‐based lifting/tightening or present safety risks if targeted. Accurate anatomical assessment guided by visualization allows selection of optimal transducer depths and treatment density, critical for maximizing treatment efficiency, safety, and patient satisfaction.

#### 3.1.3 Patient Expectation Management

Patient expectations are a critical determinant of treatment success and patient satisfaction. The panel emphasized that effective patient management begins with clear and transparent communication with patients on expected outcomes, the treatment experience, and posttreatment course. Patients should be informed that although MFU‐V can achieve measurable improvements in laxity, these effects can be subtle and may appear gradually. Patients should be advised that individual treatment response varies depending on biological factors like collagen remodeling capacity and baseline tissue quality. In some cases, optimal outcomes may require maintenance treatments and/or adjunctive therapies.

Informing patients about possible discomfort and how it will be managed is an important component of patient care and contributes to treatment satisfaction. Pain management strategies should be tailored according to patient tolerance and anatomical areas being treated, and may include topical anesthetics, oral or inhalational analgesics, or injectable local anesthetics. In the experience of several panel members, same‐day treatment of multiple regions (e.g., abdomen and arms) is feasible and offers greater time efficiency without compromising safety.

### 3.2 Treatment of the Abdomen

Abdominal skin laxity is a common concern influenced by factors such as age, weight fluctuations, and/or pregnancy. In postpartum women, changes in the abdominal skin, including laxity, wrinkling, and striae, may persist despite weight loss or return of muscle tone [17]. Although surgical options such as abdominoplasty are effective, patients may favor noninvasive alternatives due to reservations about the inherent risks of surgery and/or because abdominoplasty is generally not recommended for those planning future pregnancies.

#### 3.2.1 Clinical Evidence

Three clinical studies involving 54 participants consistently demonstrated positive outcomes with MFU‐V for abdominal laxity, as reported by both physicians and patients (Table 1) [10, 11, 12]. Based on the available evidence and their extensive clinical experience, the panel affirmed MFU‐V as a reliable noninvasive option for improving abdominal laxity in suitable candidates (Table 2). Improvements are gradual but usually discernible within several weeks of treatment, depending on factors such as age and tissue quality. After maximal results are achieved, these are often sustained for 6 months or longer, contributing to high patient satisfaction.

#### 3.2.2 Recommended Treatment Regimen

**FIGURE 1:**
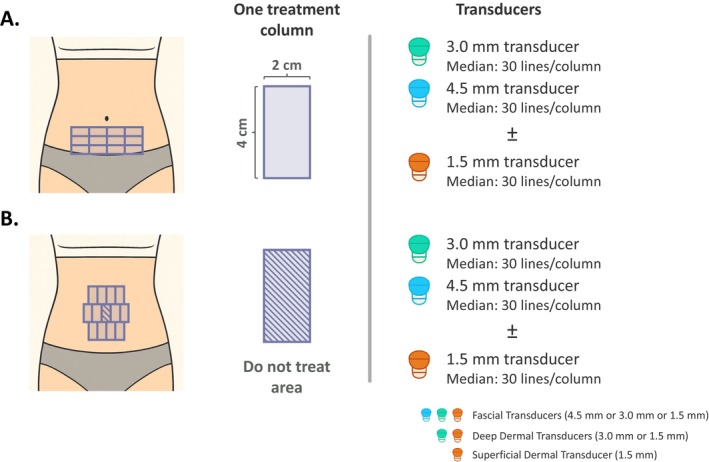
Treatment maps for the (A) lower abdomen and (B) the peri‐umbilicus region. Treatment zones are delineated using 2 × 4 cm columns, corresponding to the Ultherapy PRIME transducer. Multi‐depth treatment is recommended for both regions, with transducer selection guided by real‐time ultrasound visualization. Both the lower abdomen (A), defined as the area bounded by the umbilicus, hip bones, and pubic area, and the peri‐umbilical region (B), encompassing the area immediately surrounding the umbilicus, are commonly treated using a 12‐column grid. The 4.5 and 3.0 mm transducers are recommended for treating the superficial fascia and retinaculum cutis, respectively. If supported by ultrasound visualization findings and clinical judgment, the 1.5 mm transducer may additionally be used to target the deep dermis in cases of superficial laxity or crepiness, particularly in the peri‐umbilical area. A median of 30 lines is delivered per column per transducer. The umbilicus itself is designated as a no‐treatment zone.

Based on available clinical evidence, two treatment areas have been identified for the abdomen: (i) lower abdomen (the area bounded by the umbilicus, hip bones, and pubic area; Figure 1A) and (ii) peri‐umbilical region (the region immediately surrounding, but excluding, the umbilicus; Figure 1B). Given the tissue profile in these anatomical regions, multi‐depth treatment is considered optimal, with panelists emphasizing that transducer selection should be guided by findings from real‐time visualization. Most panelists use the 4.5 and 3.0 mm transducers to target the superficial fascia and retinaculum cutis, respectively, and optionally the 1.5 mm transducer to target the deep dermis for superficial laxity or crepiness, such as in the peri‐umbilical region.

Extended discussions among panel members showed that the number of lines commonly used in practice varied substantially. It was noted that this reflects individual variation in patients' tissue profile, body size, and aesthetic needs, which can be considerable. In proposing a more standardized approach to guide both experienced and less‐experienced operators, the panel sought to harmonize common clinical practice with recommendations based on specific clinical studies, as outlined in the MFU‐V device's Instructions for Use (IFU; Ulthera System, IFU No. 1015975IFU) (Table 3). The proposed approach is therefore based on median numbers of treatment lines, distributed across 2 × 4 cm rectangular columns, which approximates the shape/size of the MFU‐V transducer's treatment area. This corresponds to the area covered by two adjacent 2.5 × 2.5 cm squares, as per the sample schema in the product IFU (Ulthera System, IFU No. 1015975IFU). For both the lower abdomen and peri‐umbilical region (excluding the umbilicus), a 12‐column grid is recommended, with a total median of 720 lines, based on the assumption of a dual‐depth treatment strategy (Figure 1; Table 3). Energy settings of 2–4 may be used, with adjustments made based on patient comfort. In both regions, if three transducers are used, energy delivery should be distributed across all available depths, tailored to patients' anatomical characteristics and treatment requirements of each tissue plane.

**TABLE 3:** Recommended treatment regimen for the abdomen.

Site(s)	Recommended number of 2 × 4 cm rectangular columns^a^	Transducer^c^	Lines per transducer	Total lines per region^d^	Energy level range	Recommended energy level^e^
Abdomen						
Lower abdomen	12^b^	DS 4–4.5 DS 7–3.0 [DS 10–1.5]^c^	30	720	1–4	2–4
Peri umbilicus	12^b^	DS 4–4.5 DS 7–3.0 [DS 10–1.5]^c^	30	720	1–4	2–4

^a^ Treatment areas are represented with rectangular columns that correspond to the Ultherapy PRIME Transducer. With reference to the schema shown in the product IFU, two squares are roughly equivalent to one rectangular column.

^b^ The number of columns may be increased depending on the length and width of the abdomen.

^c^ Real‐time visualization should be used to guide transducer selection (1.5, 3.0, or 4.5 mm) to match the depth of the target tissue layer (dermis or superficial fascia) in the individual patient. A dual‐depth approach is most commonly applied. If supported by tissue visualization findings and clinical judgment, the option of a third transducer depth may be helpful in selected cases. The 1.5 mm transducer is most commonly used in the presence of skin crepiness.

^d^ Median recommended number of treatment lines, assuming implementation of a dual‐depth approach. The number of lines may be increased or decreased depending on patient profile (e.g., BMI, body size) and needs.

^e^ Energy level may be adjusted based on patient comfort.

### 3.3 Treatment of the Upper Arm

Upper arm laxity is a common aesthetic concern associated with aging and significant weight loss, with laxity in the posterior arm contributing to the characteristic “bat wing” appearance. Surgical treatment is rarely considered due to the potential for scarring and extended downtime [18]. MFU‐V offers a noninvasive alternative that entails minimal recovery time.

#### 3.3.1 Clinical Evidence

Five clinical studies (three for the anterior upper arm and two for the posterior upper arm) involving 113 participants reported consistent improvements in upper arm laxity following MFU‐V treatment, supported by clinician‐assessed and patient‐reported outcomes (Table 1) [12, 13, 14, 15, 16]. Panelists endorsed MFU‐V as an effective noninvasive option for addressing upper arm laxity (Table 2). As noted for the abdomen, treatment effects increase progressively, and once maximal improvement is reached, effects can last for at least 6 months.

#### 3.3.2 Recommended Treatment Regimen

**FIGURE 2:**
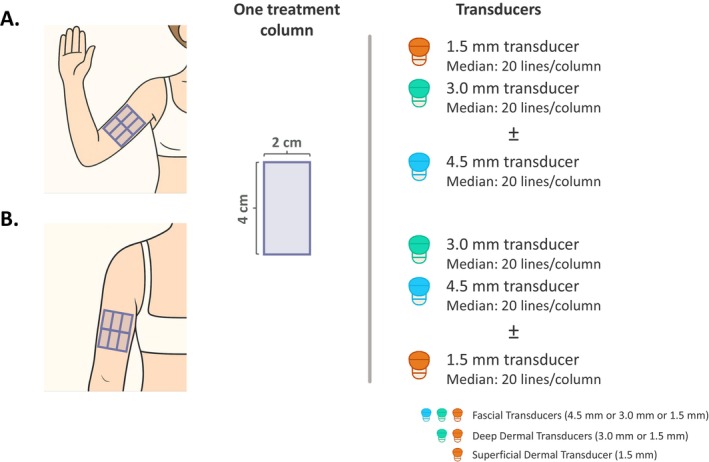
Treatment map for the (A) anterior and (B) posterior upper arm. Treatment zones are delineated using 2 × 4 cm columns, corresponding to the Ultherapy PRIME transducer. Treatment is targeted to the middle third of the upper arm, avoiding the proximal and distal thirds (i.e., closer to the shoulder and elbow), and depth targeting should be guided by real‐time ultrasound visualization. The anterior region (A), defined as the front‐facing portion of the upper arm when the arm is raised to shoulder height, the elbow is bent at a 90‐degree angle, and the palm is facing forward, is typically treated using 1.5 and 3.0 mm transducers, with 4.5 mm used selectively based on sufficient depth. The posterior region (B), defined as the back of the upper arm when the arm is relaxed and positioned alongside the body, is treated using 3.0 and 4.5 mm transducers, with occasional use of 1.5 mm for superficial crepiness. Each region is treated using a six‐column grid per arm, with a median of 20 lines delivered per column per transducer.

The two treatment regions of the upper arm are: (i) the anterior upper arm (i.e., the front‐facing portion of the upper arm when the arm is raised laterally to shoulder height, elbow bent at a 90° angle, palm facing forward; Figure 2A); and (ii) the posterior upper arm (i.e., the back of the upper arm when the arm is relaxed and positioned alongside the body; Figure 2B). For both regions, treatment should be limited to the middle third of the upper arm (between the axilla and the elbow), avoiding deeper tissues where major neurovascular structures are located.

The panel emphasized the importance of real‐time visualization to ensure accurate depth targeting. Depending upon the patient's arm profile (tissue layer distribution and composition), a dual‐depth treatment approach (typically 3.0 mm and either 1.5 or 4.5 mm) is commonly used. If supported by tissue visualization findings and clinical judgment, the option of a third transducer depth may be helpful in select cases. Panelists emphasized caution when determining treatment depths for the anterior arm as the superficial and deep fascia lie relatively close to the skin surface. Here, the 1.5 and 3.0 mm transducers are most commonly used, while the 4.5 mm transducer is applied only when sufficient depth can be visualized. In contrast, the posterior arm contains a thicker lamellar layer and deeper superficial fascia [19, 20], supporting the use of both 4.5 and 3.0 mm transducers to address soft tissue laxity. Panelists generally agreed that the 1.5 mm transducer is less frequently used in this region, where skin crepiness is less commonly observed, but may be helpful to address superficial crepiness in older patients.

For both the anterior and posterior upper arms, a total median of 240 lines is recommended per treatment region per arm, delivered across a 6‐column grid to each region (Figure 2; Table 4). An energy level of 4 is most commonly applied, with adjustments made based on patient tolerance. The total number of lines can be modified depending on the patient's profile (e.g., body size, BMI) and overall treatment goals.

**TABLE 4:** Recommended treatment regimen for upper arms (per arm).

Site(s)	Recommended number of 2 × 4 cm rectangular columns^a^	Transducer^b^	Lines per transducer	Total lines per region^d^	Energy level range	Recommended energy level^e^
Arms						
Anterior arm	6	DS 10–1.5 DS 7–3.0 [DS 4–4.5]^c^	20	240	1–4	4
Posterior arm	6	DS 7–3.0 DS 4–4.5 [DS 10–1.5]	20	240	1–4	4

^a^ Treatment areas are represented with rectangular columns that correspond to the Ultherapy PRIME Transducer. With reference to the schema shown in the product IFU, two squares are roughly equivalent to one rectangular column. The number of rectangular columns can be adjusted based on patient‐specific factors, including arm length and size.

^b^ Real‐time visualization should be used to guide transducer selection (1.5, 3.0, or 4.5 mm) to match the depth of the target tissue layer (dermis or superficial fascia) in the individual patient. A dual‐depth approach is most commonly applied. If supported by tissue visualization findings and clinical judgment, the option of a third transducer depth may be helpful in selected cases.

^c^ DS 4–4.5 is rarely used on the anterior arm, as the fascia are typically more superficial. If the fascia cannot be visualized with DS 4–4.5, consider applying an additional 30%–40% of lines using DS 7–3.0 to achieve comparable transducer coverage.

^d^ Median recommended number of treatment lines, assuming implementation of a dual‐depth approach. The number of lines may be increased or decreased depending on patient profile (e.g., BMI, body size) and treatment needs.

^e^ Energy level may be adjusted based on patient comfort.

### 3.4 Considerations Influencing Treatment Regimen

The panel emphasized that treatment should be individualized based on anatomical and patient‐specific factors. Key considerations for arm and body treatments include the degree and distribution of tissue laxity, body size and proportions (e.g., abdominal surface area or arm circumference), subcutaneous fat thickness, and skin quality. Patient characteristics such as age, BMI, and history of weight loss may influence whether superficial or deeper tissue layers should be prioritized. For example, older patients or those with a history of substantial weight change may present with crepey skin that warrants the use of superficial transducers. Gender was not identified by the panel as a factor requiring modifications to treatment protocols. Additional considerations highlighted by panelists include patient‐specific treatment goals, tolerance for discomfort, budget, and willingness to undergo multiple treatment sessions.

### 3.5 Safety Considerations

Across the various body areas addressed in clinical studies, MFU‐V demonstrated a favorable safety profile with no serious or unexpected adverse events reported (Table 1) [10, 11, 12, 13, 14, 15, 16]. The most frequently observed events were transient erythema, edema, localized tenderness, and procedural discomfort, which typically resolve within several days to approximately 2 weeks. Bruising was uncommon and, when present, was often associated with local anesthesia injections rather than the MFU‐V procedure itself. Panelists highlighted that safe and effective treatment depends on the practitioner's knowledge of anatomy and the use of real‐time ultrasound imaging to guide depth targeting and avoid off‐target energy delivery (Table 2). Special caution is advised in patients with low BMI or those with significant weight loss, where tissue depth profiles may be altered following the loss of adipose tissue. In the arms, critical neurovascular structures or joint capsules, such as those near the axilla and olecranon, require careful energy placement to avoid neurovascular injury. For the abdomen, special caution is advised in patients with a history of hernia in the abdominal region, prior surgical interventions (e.g., liposuction, abdominoplasty, or C‐section), abdominal piercings, or low BMI, all of which may alter tissue integrity and depth predictability. In these cases, real‐time ultrasound visualization is especially important to promote precise and safe treatment delivery.

### 3.6 Emerging Body Indications

Further to the newly cleared body areas, MFU‐V may offer a safe and effective option for addressing skin and soft tissue laxity in additional regions, including the knees and buttocks. Three studies (*n* = 44) evaluated treatment of the knees, and two studies (*n* = 29) assessed the buttocks. In both areas, improvements were clinically meaningful, supported by physician‐reported and patient‐reported outcomes (Table 1) [12, 15, 21, 22]. These findings support the expanding role of MFU‐V in treating additional body regions, for which regulatory approval or clearance is already available in certain countries [23]. The panel recognized the potential of MFU‐V in these regions while emphasizing the need for further research and experience to establish standardized treatment protocols, including optimal transducer depth, line density, and treatment intervals, for existing and emerging applications (Table 2).

## 4 Conclusion

This global expert consensus supports the safety and efficacy of MFU‐V in improving skin and soft tissue laxity in the abdomen and upper arms based on emerging clinical evidence and real‐world experience. These expert consensus recommendations, grounded in published data and practitioner expertise, are specific to the use of MFU‐V in body indications and are intended to guide operators in delivering safe, consistent, and effective treatment. Reproducible clinical outcomes depend on adherence to recommended treatment principles, notably effective use of real‐time ultrasound visualization, and an intrinsically patient‐centered approach that includes careful selection, expectation management, and anatomically guided tailoring of treatment. When applied appropriately, MFU‐V demonstrates a favorable safety profile with good tolerability across patient types and body regions. Currently, the evidence for MFU‐V in body indications is limited to smaller prospective studies, including a number of randomized controlled studies. As clinical use broadens, further large‐scale research and continued accumulation of published clinical experience will be valuable to strengthen and refine these recommendations, as well as to support protocol optimization in emerging treatment areas.

## Author Contributions

All authors participated in a series of virtual meetings, in which discussions were informed by a review of supporting peer‐reviewed publications and clinical studies. F.L. and V.V. contributed foundational research that informed the discussions. All authors contributed to the development of the consensus statements and recommendations, and participated in the writing, review, and editing of the manuscript for important intellectual content. All authors have read and approved the final manuscript.

## Ethics Statement

The authors confirm that the ethical policies of the journal, as noted on the journal's author guidelines page, have been adhered to. No ethical approval was required as this is a review article with no original research data.

## Conflicts of Interest

Dr. Lin serves as a speaker and investigator for Merz Aesthetics, AbbVie, L'Oréal, and BTL. Dr. Vachiramon serves as a speaker for Merz Aesthetics, Beiersdorf, L'Oréal, Vaim Global, AbbVie, Galderma, LG Chem, and Leo Pharma, and serves as an investigator for Merz Aesthetics, AbbVie, Vaim Global, Beiersdorf, and L'Oréal. Dr. Casabona serves as a consultant for Merz Aesthetics, DermapenWorld, and FillMed. Dr. Gonzaga serves as a speaker and consultant for Merz Aesthetics. Dr. Pavicic serves as a consultant and speaker for Merz Aesthetics and Advanced Aesthetic Technologies, and as an investigator for Merz Aesthetics, AbbVie, Advanced Aesthetic Technologies, and LG Chem. Dr. Spada serves as a speaker and consultant for Merz Aesthetics. Dr. Fabi serves as a consultant, investigator, and speaker for Merz Aesthetics, Galderma, AbbVie, and Revance. All authors received honoraria from Merz Aesthetics for participation in the meetings.

## Data Availability

Data sharing not applicable to this article as no datasets were generated or analyzed during the current study.
